# Male-lure type, lure dosage, and fly age at feeding all influence male mating success in Jarvis’ fruit fly

**DOI:** 10.1038/s41598-020-72209-x

**Published:** 2020-09-14

**Authors:** Suk-Ling Wee, Anthony R. Clarke

**Affiliations:** 1 Department of Biological Sciences and Biotechnology, Faculty of Science and Technology, Universiti Kebangsaan Malaysia, 43600 Bangi, Selangor Malaysia; 2 School of Biology and Environmental Science, Queensland University of Technology (QUT), GPO Box 2434, Brisbane, Qld 4001 Australia

**Keywords:** Ecology, Physiology, Chemistry

## Abstract

Males of certain Dacini fruit flies are strongly attracted to, and feed upon, plant secondary compounds such as methyl eugenol, raspberry ketone and zingerone. The consumed lure is generally found to induce physiological and behavioural changes that enhance the mating performance of lure-fed males. Male *Bactrocera jarvisi* respond strongly to zingerone from a young age, but only weakly respond to raspberry ketone. We hypothesized that this selective lure-response would be reflected in the physiological importance of the lure to the fly. We found that zingerone feeding by young males resulted in significantly greater mating success in competitive mating trials with lure-deprived flies, but the mating advantage was lost in older males. Lure dosage had a significant effect on the duration of the mating advantage, for example when fed 20 µg of zingerone, the advantage lasted only 1 day post-feeding, but when fed of 50 µg zingerone the advantage lasted 7 days. Raspberry ketone feeding did not confer any mating advantage to males except at one dosage (50 µg) for 1 day after feeding. When given a choice, *B. jarvisi* females preferred to mate with zingerone-fed versus to raspberry ketone-fed males. This study revealed lure, dosage and age of fly at time of lure administration are all important factors for maximising lure-enhanced fruit fly mating performance. These findings contribute to a better theoretical understanding of the evolution of fruit fly-lure interactions and may help improve fruit fly pest management via the Sterile Insect Technique through semiochemical-mediated enhancement of sterile male mating performance.

## Introduction

Fruit and vegetable infestation by fruit flies of the genera *Bactrocera* Macquart and *Zeugodacus* Hendel (Diptera: Tephritidae) is an ongoing problem, continuously threatening the biological and economic viability of horticultural industries across Africa, Asia and the western Pacific^1^. Monitoring and control of these fruit flies relies heavily on the usage of a small group of plant-derived secondary metabolites, of which the best known are methyl eugenol and raspberry ketone^2,3^. These chemicals are generically referred to in the fruit fly literature as the ‘male lures’, as they attract only males except under highly unusual circumstances^4^. When mixed with a toxicant, the male lures are ideal for fruit fly monitoring and attract-and-kill control because they are attractive at very low concentrations^5,6^.

The degree of male fruit fly response to male lures is associated with age and sexual maturation, but the pattern is not consistent across species^7–11^. For instance, age-related lure response of male *B. dorsalis* (Hendel) was concomitant with the onset of sexual activity^7^, but for the closely related *B. carambolae* Drew & Hancock male lure response began after the commencement of sexual activity^8^.

Male attraction to lures at a younger age, particularly before the commencement of sexual activity, is a highly desirable trait for fruit fly pest management as this enables male flies to be trapped and killed before they reach sexual maturation, so more effectively reducing population growth. Unfortunately, only a handful of the fruit fly species studied to date show strong attraction to male lures while still sexually immature. These are *B. umbrosa* (Fabricius) to methyl eugenol^9^, *B. correcta* (Bezzi) to beta-caryophyllene^10^ and *B. jarvisi* to zingerone (Tryon)^11^. In contrast to species where lure response is associated with male sexual maturation, the reason immature male fruit flies respond to lures is not well understood.

For fly species which consume male lures at or near sexual maturity, the most common lure effect is that the ingested lures are sequestered and stored, in either an unaltered or modified state, in the male pheromone glands prior to release as part of the male pheromone during courtship. Such pheromones are more attractive to females and lead to greater male mating success^9,12–16^. In *B. dorsalis*, males received a mating advantage that lasted for 35 days after consumed methyl eugenol^12^. Such chemically-mediated mating enhancement is highly desirable for increasing irradiated sterile male fruit fly mating performance in the Sterile Insect Technique^17,18^.

However, as research is conducted on a greater range of species, it is becoming clear that the lure effect on mating success varies between fruit fly species^13,15^ and between lures^10,19^. There were also reports that lure consumption has negligible effect on mating competitiveness, such as methyl eugenol for *B. cacuminata* (Hering)^20^ and zingerone for *Z. cucurbitae* (Coquillet)^21^.

Rabiatul and Wee^16^ recently demonstrated that the amount of zingerone consumed by a male *Z. tau* (Walker) contributed to differences in the mating success of those males. This observation may be important in helping to explain at least some of the divergent results seen in the fruit fly lure literature, but the effect needs wider testing to see if the results apply to other species. For example, sexually immature *B. tryoni* (Froggatt) adults experimentally fed on four concentrations of raspberry ketone mixed with sugar (this species normally only responds to the lure at sexual maturity) all matured more rapidly and exhibited an early mating advantage over lure-deprived males, but there was no treatment effect of lure concentration^22^. Additionally, the lure induced mating advantage was not maintained after 10 days of age. The differences in results between Rabiatul and Wee^16^ and Akter et al.^22^ emphasises the need for a greater number of studies on both lure concentration and fly age effects, as well as the need for species by species studies.

*Bactrocera jarvisi* (Tryon) (Diptera: Tephritidae) is an endemic, polyphagous Australian fruit fly of known pest status^23^. In the field, male *B. jarvisi* are strongly attracted to zingerone and very weakly attracted to raspberry ketone^24^. In the laboratory, dose–response curves demonstrate that the species is approximately 1,500 times more responsive to zingerone than raspberry ketone: the species is also attracted to zingerone at a much younger age than to raspberry ketone^11^. The carbonyl group, methoxy group, and phenolic of zingerone are considered the key structural elements which make that chemical so attractive to *B. jarvisi*^25^. However, despite detailed knowledge on the relative attractiveness of *B. jarvisi* to raspberry ketone and zingerone, what role, if any, the two chemicals play in sexual selection in the species is not known.

Here we use *B. jarvisi* as a model fruit fly to address the effects of insect age, lure type and lure dose on male mating success. The work builds on the findings of Wee et al.^11^ which showed that *B. jarvisi* males are first attracted to zingerone at a much younger age than to raspberry ketone, and that the species’ optimum response to zingerone was achieved at ca. 11 day-old before 50% of the population had mated at 16–17 day-old. Therefore, we first investigate whether zingerone feeding at a younger (at or below 2 week-old) and older age (between 3 and 4 week-old) affects the outcome of mating trials when flies are sexually mature. This experiment is similar to that done for raspberry ketone fed *B. tryoni* by Akter et al.^22^, but in that system the fly does not naturally respond to the lure when sexually immature. Second, due to the disparity of responsiveness of *B. jarvisi* males to zingerone and raspberry ketone, and the low concentration of these phytochemicals present in natural sources^26,27^, we tested the influence of low (as found in nature), medium and high (as used in trapping) doses on the mating success of zingerone- and raspberry ketone-fed males in comparison to the lure-deprived males. Finally, we tested the prediction based on the sensory preference of *B. jarvisi* males towards zingerone, that zingerone-fed males would receive greater direct mating benefits (if they occurred) than would raspberry ketone-fed males in a competitive mating arena.

## Results

### Experiment 1: Effects of age on mating competitiveness of zingerone-fed males

The mean mating success of older (21–28 day) zingerone-fed males (40.0 ± 5.5%) was not signficantly different to zingerone-deprived males (35.0 ± 5.0%) (Mann–Whitney U = 59.5, n_1_ = n_2_ = 12, *P* = 0.46). However, young zingerone-fed *B. jarvisi* males (11–15 day) achieved signficantly more matings (62.5 ± 2.5%) than young zingerone-deprived males (27.5 ± 3.7%) (Mann–Whitney U = 0.0, n_1_ = n_2_ = 8, *P* < 0.001).

### Experiment 2: Effects of lure dose on mating competitiveness of lure-fed males

#### Zingerone

Zingerone feeding significantly increased male mating success in *B. jarvisi*, but inconsistently with dosage. At the lowest dosage (20 µg) the mating advantage was seen at day 1 after feeding (1 DPT), but not at 3 DPT. At the medium dosage (50 µg) a significant effect was seen at 1, 3 and 7 DPT, but not at 14 DPT. At the highest dosage (100 µg) there was a significant mating benefit of lure feeding at 3 DPT, but not at 7 DPT nor, unexpectedly, at 1 DPT (Table 1).

**Table 1:** Mean (± 1 sem) percentage mating success of zingerone (ZN)- or raspberry ketone (RK)-fed *Bactrocera jarvisi* males in competitive mating trials with lure-deprived males at different lure concentrations and different day post treatment (DPT).

Lure	Lure dose per fly (µg)	DPT	n	Mean mating success (%)		*t/U*	*df*	*P*
				Lure-fed	Lure-deprived			
ZN	20	1	12	60.0 ± 3.5	31.67 ± 4.6	*U* = 30.5	–	0.004
		3	10	48.0 ± 4.4	42.0 ± 4.7	*t* = 0.930	18	0.365
	50	1	8	77.5 ± 4.5	15.0 ± 6.3	*U* = 0	–	< 0.001
		3	8	52.5 ± 6.1	15.0 ± 5.9	*t* = 4.114	14	0.001
		7	8	67.5 ± 6.4	30.0 ± 6.5	*t* = 3.84	14	0.002
		14	9	35.6 ± 6.5	24.4 ± 4.4	*t* = 1.268	16	0.223
	100	1	16	46.3 ± 4.0	48.8 ± 4.1	*U* = 106.5	–	0.807
		3	7	62.9 ± 6.8	28.6 ± 4.0	*t* = 4.313	12	0.001
		7	8	50.0 ± 6.5	40.0 ± 6.5	*U* = 24	–	0.442
RK	20	1	8	30.0 ± 5.4	40.0 ± 8.5	*t* = 1.016	14	0.327
		3	8	35.0 ± 3.1	40.5 ± 6.1	*t* = 1.698	14	0.112
	50	1	8	55.0 ± 5.0	20.0 ± 5.3	*t* = 4.532	14	< 0.001
		3	8	37.5 ± 5.9	21.4 ± 7.6	*t* = 0.888	14	0.390
		7	8	32.5 ± 7.5	17.5 ± 5.9	*t* = 1.551	14	0.143
	100	1	8	32.5 ± 5.3	45.0 ± 3.3	*U* = 16	–	0.105
		3	6	46.7 ± 8.4	43.3 ± 9.5	*t* = 0.269	10	0.793

Males were sexually mature at a previously determined optimum age for mating, of between 11 and 16 days. Each replicate consisted of five lure-fed and five lure-deprived males competing for five virgin females. Student’s *t*-test was used for comparison for normally distributed data, while Mann–Whitney U test was used when normality of data was not met.

#### Raspberry ketone

For nearly all treatments raspberry ketone had no significant effect on male mating competitiveness. Males fed the medium dosage (50 µg) had a significant mating advantage over unfed males at day 1 after feeding, but not thereafter. Flies fed low and high dosages of raspberry ketone had no significant mating advantage over lure-deprived males on any day (Table 1).

### Experiment 3: Effects of zingerone and raspberry ketone feeding on male mating competitiveness

At 1 day post feeding, when zingerone-fed and raspberry ketone-fed males were released at the same time to compete for females, the mean mating success of zingerone-fed males (55.6 ± 6.5%) was significantly higher than that of raspberry ketone-fed males (17.8 ± 5.2) (Mann–Whitney U = 4.000, n_1_ = n_2_ = 9, *P* = 0.001).

## Discussion

Building on our previous findings on the olfactory sensory sensitivity and bias of *B. jarvisi* males towards zingerone over raspberry ketone^11^, this study further reveals that fruit fly age, lure type and lure dose all impact on lure-mediated fruit fly mating success. The present results also clearly indicate that zingerone plays a more prominent role than raspberry ketone in *B. jarvisi* sexual selection. The following discussions addresses each of the three key elements of the experiments, i.e. fly age, lure dosage, and lure type, and then relates all findings to their importance for pest management.

The outcome of competitive mating trials for young (11–15 day-old) and older (21–28 day-old) *B. jarvisi* males after consuming zingerone was markedly different. The mating success of the young zingerone-fed males was significantly greater than those of the young zingerone-deprived males; but when flies were approximately 10 days older no such mating advantage occurred. *Bactrocera jarvisi* males respond to zingerone from as young as 3 day-old when still sexually immature. At 7 day-old, when only 0.5% of males in a population had attained sexually maturity, more than 50% of males had responded to the lure; and fastest lure response occurred at 11 days of age, approximately a week before 50% of the male population had sexually matured^11^. Combined, the current and earlier data sets show the importance of zingerone to very young and young *B. jarvisi* as they are sexually maturing and newly sexual mature, but also demonstrate that the lure appears to have lesser importance for older males.

Zingerone displays pharmacological properties of enhancing growth and antioxidant activity^28,29^ and was shown to act as a metabolism enhancer in the fruit fly *B. tryoni*^30^. Zingerone feeding was also shown to increase mating success of fruit flies by increasing male courtship activity in *B. tryoni*^30^ and *Z. tau*^16^ and the attractiveness of the male pheromone in *Z. tau*^16^. Given the direct benefits that male fruit flies may receive upon zingerone feeding, it is perhaps not unexpected that young *B. jarvisi* males would actively forage for the lure to increase their maturation rate and to enhance mating competitiveness. However, the reason why zingerone feeding did not offer any significant mating advantage to the older *B. jarvisi* is less clear. In *B. tryoni*, males received a mating advantage from feeding on zingerone^14^, but this was subsequently shown to be due the metabolism enhancement properties of the lure^30^, and not because it enhanced the attractiveness of the male pheromone^19^. If in *B. jarvisi* there is similarly no pheromone enhancement effect, and by the age of 21 days all individuals have obtained sexual maturity, then zingerone may offer no further sexual selection benefits, so leading to the results obtained: this hypothesis will be tested. However, independent of its lack of benefit in sexual selection, *B. jarvisi* males still respond strongly to zingerone beyond 21 days^11^ and so we speculate that zingerone may play biological role(s) other than mating enhancement for older flies, such as increased immune response^29^ or more efficient energy use^31^: this warrants future investigation.

The variable lure doses offered to the male flies not only affected the outcome of the mating trials, but also the period of sexual enhancement. For example, at 20 µg (low dose), zingerone conferred significant mating advantage males at 1 DPT, but not at 3 DPT. This is presumably due to the rapid exhaustion of lure effect because of the very low dose initially supplied. However, when 100 µg zingerone (high dose) was offered, the mating success of 1 DPT males was not significantly different from that of the zingerone-deprived males, but then a significant mating advantage was recorded at 3 DPT, which was lost again by 7 days. In contrast again, when a median dose of 50 µg zingerone was offered to *B. jarvisi* males, the zingerone-fed males had significant mating advantage for at least 7 days (lost by 14 days). The underlying mechanism(s) for these complex effects is unknown, but it does reinforce the point that lure dosage is critical and may partially explain why in some studies, where lure intake of flies has not been controlled, conflicting results have been obtained (see e.g. Kumaran et al.^14^ and Inskeep et al.^21^).

Consumption of raspberry ketone by *B. jarvisi* yielded vastly different results compared to zingerone consumption. Raspberry ketone consumption at low and high doses did not confer any mating advantage to *B. jarvisi* males. At the median dose of 50 µg raspberry ketone-fed males had a significantly greater mating success than the raspberry ketone-deprived males at 1 DPT, but not at 3 and 7 DPTs. Together with the very weak attraction of *B. jarvisi* males to raspberry ketone^11^, we infer that raspberry ketone plays little role in the chemical ecology of *B. jarvisi*.

In the ever growing fruit fly lure literature, there have been an increasing number of papers which present divergent and/or conflicting results on the importance or otherwise of the male lures to *Bactrocera* and *Zeugodacus* species. For example, Rabiatul and Wee^16^ reported that the rectal gland extracts of *Z. tau* males after feeding on 50 µg zingerone attracted significantly more females than the corresponding control males, with the period of male mating advantage lasting for 7 days after initial feeding. In contrast, for *B. tryoni* and *Z. cucurbitae*, respectively, zingerone feeding conferred a male mating advantage for only 1 day^14^ or not at all^32^. While such divergent results in the literature were initially treated as experimental error or misinterpretation (see for example comments in Shelly^2^ on Raghu and Clarke^33^), increasingly there is recognition that the impacts of lures vary with species and lure type. However, this study, and the work of Akter et al.^22^ and Rabiatul and Wee^16^, identify further sources of divergence between studies even when studying the same fly and lure, i.e. fly age and lure dosage. This has significant impacts not just for the theoretical development of the field, but also for applied entomology. For the male annihilation technique, a lure-and-kill approach which uses the male lures as the attractant^34^, optimising the initial lure dosage may lead to increases in trap efficiency. For the Sterile Insect Technique, determining optimum age and lure dosage is likely to significantly impact the value of pre-release feeding of lures to sterile flies for enhancing growth and mating competitiveness^18,22^.

## Materials and methods

### Insects

*Bactrocera jarvisi* of 6–8th generation from the wild (originally reared from mango) were obtained as pupae from the Department of Agriculture and Fisheries, Brisbane, Queensland. Emerged flies were provided with artificial adult diet comprised of protein hydrolysate-sugar mixture (ratio 3:1) and water ad libitum. All flies were sex-segregated within 2 days of emergence and maintained separately in screen cages (40 × 40 × 40 cm). The insectary was maintained at 27 °C and 70% RH and received fluorescent lighting between 07:00 and 16:00 h daily in addition to natural sunlight.

### Chemicals and chemical concentrations

Zingerone (4-(4-hydroxy-3-methoxyphenyl)-2-butanone; CAS 122-48-5) and raspberry ketone (4-(4-hydroxyphenyl) butan-2-one; CAS 5471-51-2) (both > 96% purity, Sigma-Aldrich) were diluted stepwise in absolute ethanol (≥ 99.8% purity; Sigma-Aldrich) to obtain 2 µg/µL, 50 µg/µL and 100 µg/µL for fly feeding purpose. The low feeding dose at 20 µg was chosen to imitate the minimum amount of zingerone that could be found in a *Bulbophyllum* orchid flower^26,27^, a natural lure source: this concentration thus represents a level relevant to evolutionary and ecological interactions. The high feeding dose at 1 mg represents the amount of lures often used in fruit fly pest management programmes. A median dose at 500 µg was chosen as the dosage to which *B. jarvisi* most rapidly responds to both lures^11^.

### Preparation of experimental flies

Lure feeding by males was conducted in the morning between 09:00–11:30 h in a laboratory, maintained at 22–25 °C and 65–70% RH, which was isolated from the insectary. The lure dose was dispensed using a pre-calibrated glass micropipette (Drummond USA). For low dose lure feeding, an aliquot of 10 µL of a 2 µg/µL solution was pipetted onto a glass slide. After allowing time for solvent evaporation (approx. 10 s), an individual male was allowed to feed on the glass slide for 30 min. Similar procedures were followed for median dose (500 µg) and high dose (1 mg) treatments. An aliquot of 10 µL lure solution was dispensed onto a piece of 35 mm diameter filter paper placed inside a disposable 35-mm Petri dish and offered to 10 male flies for 30-min feeding. Our experimental assumption was that all lure dispensed would be consumed, equally, by the males within 30 min of feeding duration, thus creating treatments of 20 µg, 50 µg, and 100 µg lure per fly for low, median and high dose exposure: the control males were not lure exposed. After lure feeding, the treated male was then provided with sugar-hydrolysate diet and water ad libitum until required for trials, which was 1 day later unless otherwise stated.

To differentiate between lure-fed and lure-deprived males, the thoracic dorsum of males from each category were marked with non-toxic paint. To eliminate bias, colour markings of lure-fed and lure-deprived males were alternated between replication.

### Mating competition trials

Effects of lure consumption on *B. jarvisi* male mating success were evaluated under different age categories, lure and lure dosages in screen-cage (40 × 40 × 40 cm) mating trials according to procedures described by Kumaran et al.^14^. To assess the effects of zingerone or raspberry ketone consumption on male mating competitiveness, five lure-fed and five lure-deprived virgin males were allowed to compete for five virgin and sexually mature females (14–21 days after emergence). All fly groups, contained in separate screen cages with food and water, were placed in the experimental site 2–3 h before the onset of dusk (the mating time in this species) to allow for acclimatization. At 17:00 h, males were released first into the mating cage to allow time for male territory establishment, followed by females at 30 min later. Food and water were removed before mating trials commenced. One hour after sunset, mating pairs were carefully coaxed into individual glass vials under low light intensity from a torch screened with red lantern paper. The identity of males for each pair was recorded noted based on their thoracic colour marking.

#### Experiment 1: Effects of age on mating competitiveness of zingerone-fed males

*Bactrocera jarvisi* males first respond to zingerone at 3 days after emergence, before any mating is observed. The most rapid lure responses occur at 9–11 days of age when some flies in a population are just becoming sexually mature. Fifty percent of a population has mated by 16–17 days of age, and most mating in a population has occurred at 21 days of age^11^. Given these developmental times, we offered 20 µg zingerone to *B. jarvisi* males of two age categories, 11–15 days after emergence (= young-age treatment, flies show rapid lure response and active mating) and 21–28 days after emergence (= older-age treatment, flies show slower lure response and minimal mating). Competitive mating trials were then run 1 day after lure exposure to determine if there was any difference in the outcome of mating competition between zingerone-fed and zingerone-deprived young males, and between zingerone-fed and zingerone-deprived older males. Eight replications were run for the young-age treatment and 12 replications for the older-age treatment.

#### Experiment 2: Effects of lure dose on mating competitiveness of lure-fed males

Young and mature-age *B. jarvisi* males were each offered low (20 µg), mid (500 µg) and high doses (1 mg) of zingerone or raspberry ketone and then competitive mating trials run against control males of the same age at 1-, 3-, 7- or 14-days post treatment (DPT). The days PT each experiment was run varied depending on when the treatment effects became non-significant, as an assumption was made based on earlier research with zingerone and raspberry ketone^16,30,35^ that any biological effect of these lures is short lived and would not “reappear” after becoming non-significant.

#### Experiment 3: Effects of zingerone and raspberry ketone feeding on male mating competitiveness

Young and mature-age *B. jarvisi* males were each offered 500 µg of either zingerone or raspberry ketone. A day after lure feeding, competitive mating trials were run between the matched-age pairs of zingerone-fed and raspberry ketone-fed males.

### Statistical analysis

All mating data obtained were subjected to Normality test (Shapiro–Wilk) and Equal Variance test. When the normality and equal variance assumptions were met, Student’s *t*-test (two-tailed) was used to evaluate if there was any difference in the mating competitiveness between treatment and control flies, and between zingerone-fed and raspberry ketone-fed males (experiment 3). Non-parametric alternative Mann–Whitney Rank Sum test was used when normality and equal variance assumptions were not met. All analysis was performed using Sigma Plot 12.0, with α set at P = 0.05. Results are presented as means ± 1 sem.

### Ethical approval

This article does not contain any studies with human participants or animals (vertebrates) performed by any of the authors.

## Data Availability

Data are stored at Department of Biological Science and Biotechnology, Universiti Kebangsaan Malaysia and are available from the lead author on request.
